# A miniaturized endocardial electromagnetic energy harvester for leadless cardiac pacemakers

**DOI:** 10.1371/journal.pone.0239667

**Published:** 2020-09-28

**Authors:** Nicolas Franzina, Adrian Zurbuchen, Andreas Zumbrunnen, Thomas Niederhauser, Tobias Reichlin, Juergen Burger, Andreas Haeberlin

**Affiliations:** 1 Department of Cardiology, Bern University Hospital, University of Bern, Bern, Switzerland; 2 Sitem Center for Translational Medicine and Biomedical Entrepreneurship, University of Bern, Bern, Switzerland; 3 ARTORG Center for Biomedical Engineering, University of Bern, Bern, Switzerland; 4 Institute for Human Centered Engineering, Bern University of Applied Sciences, Biel, Switzerland; Universitatsklinikum Wurzburg, GERMANY

## Abstract

Life expectancy of contemporary cardiac pacemakers is limited due to the use of an internal primary battery. Repeated device replacement interventions are necessary, which leads to an elevated risk for patients and an increase of health care costs. The aim of our study is to investigate the feasibility of powering an endocardial pacemaker by converting a minimal amount of the heart’s kinetic energy into electric energy. The intrinsic cardiac muscle activity makes it an ideal candidate as continuous source of energy for endocardial pacemakers. For this reason, we developed a prototype able to generate enough power to supply a pacing circuit at different heart rates. The prototype consists of a mass imbalance that drives an electromagnetic generator while oscillating. We developed a mathematical model to estimate the amount of energy harvested from the right ventricle. Finally, the implemented prototype was successfully tested during in-vitro and in-vivo experiments.

## Introduction

Over the last decades, the number of pacemaker (PM) implantations constantly increased and exceeded 1.5 million implantations annually worldwide [1]. Even though PM therapy is well-established, patients are often forced to undergo repeated surgical interventions due to PM battery depletion [2]. Re-interventions increase the risks of complications and pose an economic burden. To avoid lead-related system failures, leadless intracardial PMs have been introduced [3]. According to the manufacturer’s manual, they have an estimated autonomy of 8 to 10 years and a power consumption of ~4 μW at a 100% pacing rate of 60 beats per minute (bpm). Although revolutionizing the world of PMs, the dependency of leadless systems on batteries is a major problem: once these devices are overgrown by tissue, they cannot be explanted anymore. In addition, the battery’s volume and therefore also its capacity is strongly limited.

To avoid recurrent surgical interventions, leadless PMs need to rely on an autonomous and long-lasting energy source. Different methods that convert energy from the heart to electrical energy have been investigated. These mechanisms were developed to either harvest energy from the heart motion, pressure variations in the heart or blood stream. Piezo-electric mechanisms can be utilized to transform a small portion of the cardiac motion or the blood pressure variations into electrical energy [4–9]. They rely on a beam structure with a proof mass, which is bent in the presence of heart motion. Also electromagnetic transducers can be used to harvest kinetic energy of the heart. [10] and [11] demonstrate the use of a wristwatch mass imbalance principle coupled to a microgenerator that generates an oscillating voltage during the cardiac motion cycle. [12] also shows an endocardial energy harvesting approach that makes use of a magnet stack oscillating through coils in the presence of cardiac motion. Finally, energy may also be harvested from the blood flow inside the heart by a bi-stable beam coupled to an electromagnetic transducer [13]. These approaches suffer from important limitations such as invasive implantation [7–11], single-axis acceleration dependency [4, 5, 9, 12], risk of blood clot formation [13], too low harvested power [6] or shape and size not adapted to be implanted inside the heart [5, 8–10].

This work investigates the feasibility of endocardial energy harvesting from the ventricular heart motion by means of a miniaturized electromagnetic harvesting device. The harvester is based on an imbalanced mass coupled to a micro-generator. One of the main design criteria was that the housing of the device should not exceed the dimensions and shape of contemporary leadless PMs to guarantee catheter-based implantation and minimize the risk of side-effects due to interferences with the physiologic behaviour of the heart. For the development of a device capable of harvesting enough energy to power the electronics of cardiac pacemakers, a numerical simulation was introduced to estimate the power output in silico. Based on that, a prototype was built and extensively tested during test bench and in-vivo experiments.

## Methods and materials

### I. Harvesting principle

**Fig 1:**
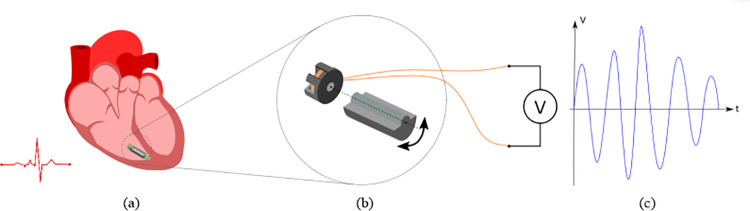
Schematic representation of the energy conversion principle: the movement of the heart during a cardiac cycle (a) makes the unbalanced half-cylindrical weight oscillating (b) and induces an electric signal In the coil of the generator coupled to its rotation axis (c).

The energy harvesting device presented is based on an electromagnetic induction principle. It uses a mass imbalance as rotor to translate the heart’s vibrations into rotation. Permanent magnets placed on the rotor induce a current flow into a coil placed on the stator. The device consists of an oscillating weight directly coupled to a micro-generator MG4.0 by KINETRON (Fig 1).

To define the dimension and material of the weight, in-silico experiments were performed varying shape and weight of the imbalance mass (see next paragraph). The harvester was developed to be implanted in the same way as contemporary leadless endocardial pacemakers–by a trans-catheter implantation approach. Therefore, external device dimensions were restricted to a maximum of 30 mm in length and the 7 mm in diameter. Using dimension that are similar to commercially available endocardial pacemakers reduces the risk to observe undesired side-effects due to the device in the heart.

### II. Mathematical model

A mathematical model has been introduced to estimate the amount of energy the mechanism can convert from specific heart motions. To investigate how the oscillating weight dimensions influence the power output, the mechanism was numerically described and analysed in MATLAB and Simulink (Mathworks Inc., USA).

**Fig 2:**
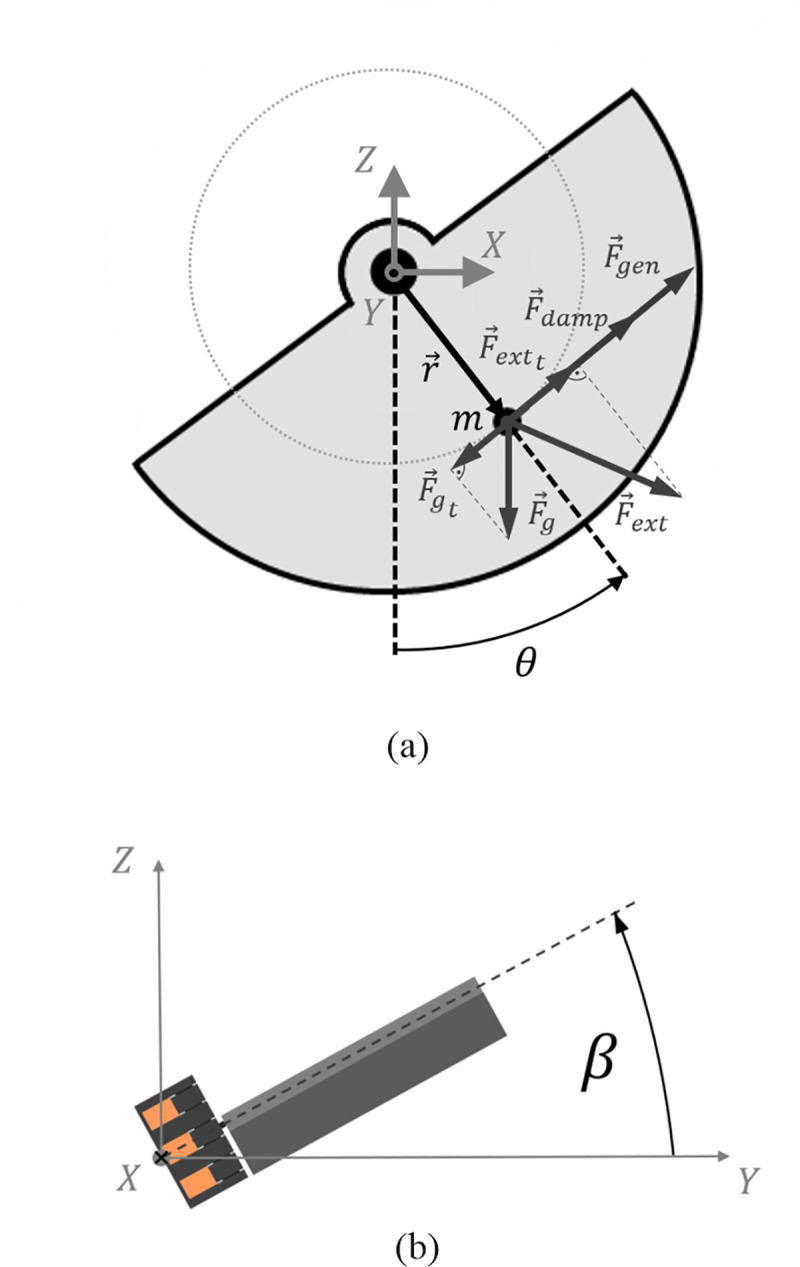
Free body diagram of the harvesting system representing a section of the imbalanced weight. The external force *F*_*ext*_, initiated by translational and rotational accelerations, causes an oscillation movement of the weight along the rotation axis (a). The mathematical model also takes into account the orientation of the pendulum compared to the horizontal XY-plane (b).

The mathematical model bases on a physical pendulum with a half-cylindrical mass rotating around the y axis. Various force components act tangentially on the circle described by the centre of mass *m* at a radius *r* (cf. free body diagram, Fig 2). According to Newton’s second law, the problem can be described by the sum of all the torques

$$\tau_{tot}=I\theta \prime \prime =\sum \tau =r\sum \vec{F}$$ (1)

The following mathematical pendulum model only considers the tangential force components. Specifically, the model accounts for the damping force

$$F_{damp}=-\mu_{d}r\theta \prime$$ (2)

where *μ*_*d*_ is the damping factor of the system and *θ*′ the angular velocity. The tangential component ${{\vec{F}}_{g}}_{t}$ of the gravity ${\vec{F}}_{g}$ acting on the weight can be described as follow:

$${F_{g}}_{t}=-mg_{m}\left(sin\theta \right)$$ (3)

where *g*_*m*_ is the gravitational acceleration acting on the pendulum.

An external acceleration (e.g. cardiac movement) is described as external force ${\vec{F}}_{ext}$ and drives the oscillating mass:

$${\vec{F}}_{ext}=m\vec{a}=m\left(\begin{array}{c} a_{X}\\ a_{Z} \end{array}\right)$$ (4)

*a*_*X*_ and *a*_*Z*_ are the components of the external acceleration $\vec{a}$ acting on the pendulum. To investigate the influence of the device orientation in the heart with respect to the external acceleration and to gravity, the tilt angle *β* was added to the model, describing the angle between the rotation axis of the pendulum and the XY-plane (cf. Fig 2B). Considering this tilting effect, the external acceleration can be described as:

$$\vec{a}=\left(\begin{array}{c} a_{X}\\ a_{Z} \end{array}\right)=\left(\begin{array}{c} a_{ext_{X}}\\ a_{ext_{Z}}cos\beta +a_{ext_{Y}}sin\beta \end{array}\right)$$ (5)

Where $a_{{ext}_{X}},\;a_{{ext}_{Y}}$ and $a_{{ext}_{Z}}$ are the component of the heart acceleration previously measured in-vivo (cf. section V). The tilt angle of the pendulum also affects the gravitational component acting on the pendulum. ${{\vec{F}}_{g}}_{t}$ of the gravity ${\vec{F}}_{g}$ acting on the oscillating weight can therefore be described as follows:

$${F_{g}}_{t}=-mg\;sin\left(\theta \right)\cos\left(\beta \right)$$ (6)

Where *g* is the gravitational constant acting on the pendulum.

The tangential component of the external force can be mathematically described by the projection of the external force onto the tangential axis $\vec{t}$, which can be obtained by rotating the radius vector $\vec{r}$ by 90° in clockwise direction:

$$\vec{t}=\left[\begin{array}{cc} 0 & -1\\ 1 & 0 \end{array}\right]\vec{r}=\left(\begin{array}{c} r\;cos\;\theta \\ r\;\sin\;\theta \end{array}\right)$$ (7)

This allows describing the tangential component of the external force

$${\vec{F}}_{ext_{t}}=m\left(proj_{\vec{t}}\vec{a}\right)$$ (8)

as well as its norm in order to find a numerical solution:

$$F_{ext_{t}}=a_{X}\sin\;\theta +a_{Z}\;\cos\;\theta$$ (9)

In addition, the numerical model considers three different torques that arise in our electromagnetic generator. The friction of the generator *τ*_*f*_ that depends on the viscous friction coefficient *μ*_*gen*_ and the angular velocity *θ*′

$$\tau_{f}=-\mu_{gen}\theta^{\prime },$$ (10)

the permanent magnet of the rotor that causes a detent torque *τ*_*det*_

$$\tau_{det}=-T_{yMax}\sin\;\theta$$ (11)

and the induced electromagnetic fields that create a torque *τ*_*em*_ according to faraday’s law of induction, considering a rotor magnet with *N*_*p*_ = 7 number of poles

$$\tau_{em}=I_{gen}\frac{d\Phi }{d\theta }=I_{gen}N_{p}\Phi_{\max}\sin\left(N_{p}\theta \right)$$ (12)

where *I*_*gen*_ is the induced current in the generator’s coil with a known load and internal resistance *R*_*L*_ and *R*_*gen*_, respectively.

$$I_{gen}=-\frac{\theta^{\prime }}{R_{L}+R_{gen}}\frac{d\Phi }{d\theta }=-\frac{\theta^{\prime }N_{p}\Phi_{\max}\sin\left(N_{p}\theta \right)}{R_{L}+R_{gen}}$$ (13)

The three torques described above were investigated by Lossec [14], who experimentally derived the constants *μ*_*gen*_, *T*_*yMax*_ and Φ_*max*_ to model this specific micro-generator.

According to Eq 1, all force and torque components can be summarized to get the total torque equation

$$\theta^{\prime \prime }=\left(r\left(F_{g}{}_{t}+F_{damp}+F_{ext_{t}}\right)+\tau_{f}+\tau_{det}+\tau_{em}\right)/I$$ (14)

where $I=\frac{1}{2}m({r_{cylinder})}^{2}$ is the moment of inertia for a half-cylinder and *θ*″ the angular acceleration of the oscillating weight. Substituting the force and torque components by the formulas 2–4 and 7–10 leads to a second-order explicit ordinary differential equation (ODE) with the angle *θ* as time-dependent variable. The given ODE can numerically be solved by classical algorithms, such as a the Runge-Kutta algorithm.

### III. Heart movement simulation platform (hexapod)

**Fig 3:**
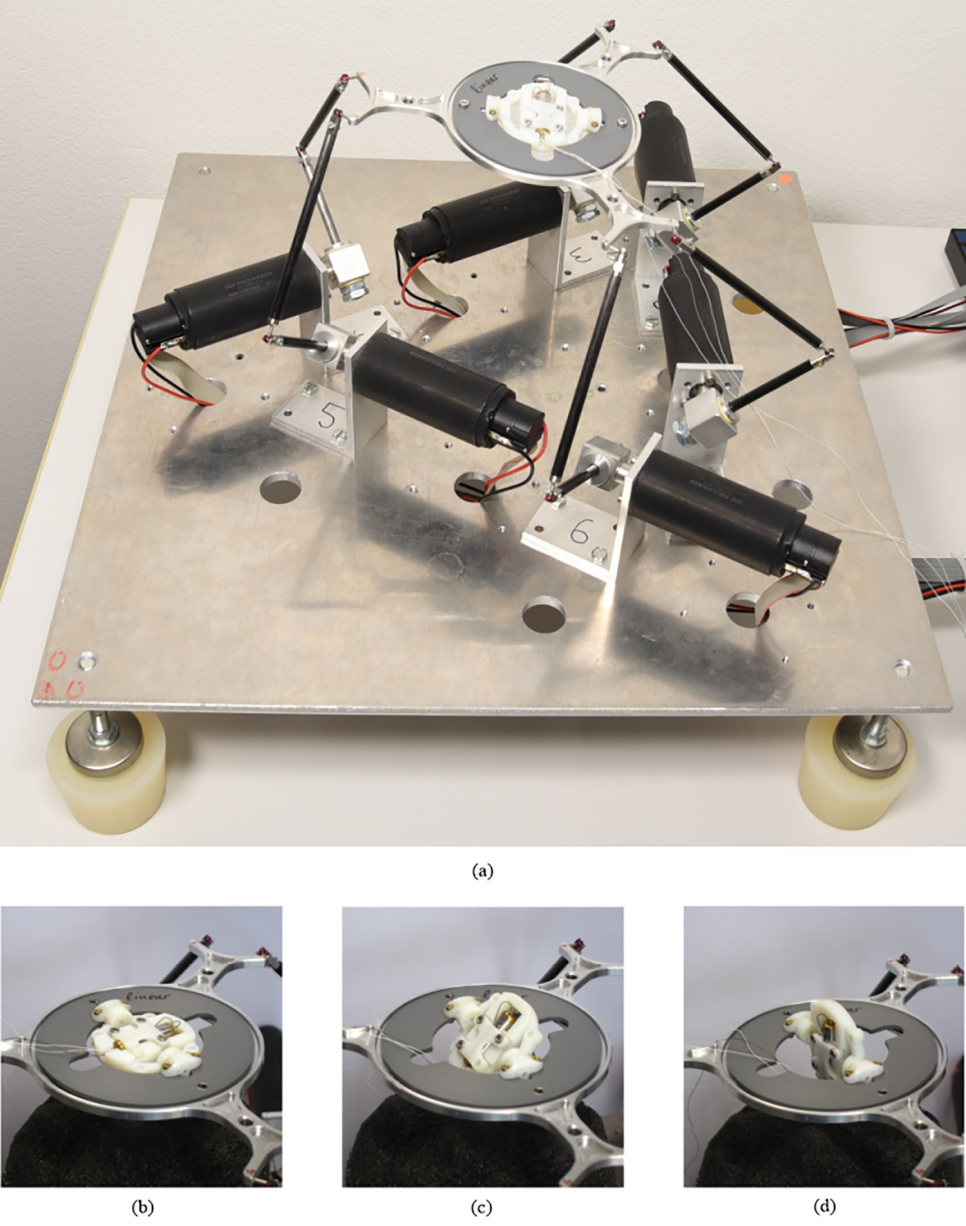
The hexapod has six articulated arms that connect the motors with the end effector platform (a). The end effector platform holds the energy harvester that can be tilted to investigate the effect of different angles (b) (c) (d).

The hexapod–also known as Stewart platform—is a custom-made robot used to mimic heart movements [15]. It provides reproducible heart motion scenarios to test the harvesting device on the bench and thereby reduce the number of animal trials necessary. The robot consists of a platform, which is driven by 6 electric motors (3272G024CR, Faulhaber, Switzerland) (Fig 3). The hexapod platform can be moved and tilted in 6 degrees of freedom, which allows the robot to move the prototype along previously acquired heart motion trajectories (cf. section V for more details on the heart motion data acquisition). This way, the effect of heart accelerations on the oscillating weight could be validated experimentally. The accuracy of the reproduced motion path always depends on the trajectory used as input for the hexapod and the median error of the platform positioning and orientation for all trajectories was 0.26 mm (IQR 0.12–0.4 mm) and 0.23° (IQR 0.11–0.35°), respectively.

### IV. Model validation

To validate the mathematical model, the damping coefficient was investigated by comparing the output power of the simulation with the one measured on the hexapod.

The mathematical model and the hexapod bench experiment were using the same heart motion trajectories as input. In both settings, the energy harvester was oriented in three different angles with respect to the gravity vector, i.e. in 35, 45 and 90 degrees. Furthermore, various lengths of the oscillating weight (L8: 8 mm, L16: 16 mm and L32: 32 mm) were tested.

The dependency on too many unknown factors of the damping coefficient *μ*_*d*_ for the differential equation makes the estimation of its value hard to determine by analytical methods. Therefore, a damping coefficient was chosen with which the simulation output would align optimally with on-bench measurements. The damping coefficient *μ*_*d*_ accounts for dry friction and air pumping in the enclosed housing, which are difficult to determine analytically. For this reason, a wide range of damping coefficients from 0.03 to 0.6 were investigated. In this range, a damping coefficient was selected that minimizes the overall estimated power error compared to the power measured during the bench experiment.

The hexapod output measurement was performed three times. The output power estimated by the mathematical model were then compared to the output power obtained by hexapod experiments. The voltage generated by the harvester was recorded with LabView (National Instruments Corporation, USA) using the data acquisition device USB-6008 (National Instruments, USA). The signals were analysed using MATLAB.

### V. Experimental set-up

The hexapod robot was used to expose the energy harvesting prototype to a realistic heart motion trajectory. The heart motion data describing three-dimension acceleration and angular velocities were processed into trajectories, which the robot is able to follow in an endless loop, starting over again every 60 seconds. A 3D printed mount held the prototype in place on the robot’s platform (Fig 3A). The device could be tilted and secured in different angular positions.

As described in more detail in [12], heart motion data were acquired by an implantable acceleration sensor during an in-vivo study of a 60kg domestic pig. The sensor recorded accelerations and angular velocities in three dimensions from the right ventricle in supine position. The data were recorded at varying heart rates and pacing locations. The first dataset corresponds to the intrinsic cardiac activity of the animal during the experiment. The second and third datasets were acquired at higher heart rates enforced by a temporary pacemaker performing overstimulation at a rate of 120, 130, 150 and 160 beats per minute (bpm).

The pacing was performed via epicardial wires sutured onto the left atrium and the left ventricular apex, respectively. In total, nine different heart motion trajectories were acquired from an animal trial and used in the experimental setup and simulation. The trajectories represent one intrinsic and four artificial heart rates and two different pacing locations (Table 1). In addition, a human trajectory dataset was extracted from magnetic resonance imaging (MRI) tagging of the entire left ventricle of a healthy 30-year-old male [10, 16]. Both subjects are assumed to be healthy.

**Table 1:** Characteristics of the In-vivo acquired trajectories reproduced by the hexapod.

Trajectory Nr.	Acquired by	Species	Pacing	Heart rate
				[bpm]
1	Accelerometer	Pig	None	90
2	Accelerometer	Pig	Atrial	120
3	Accelerometer	Pig	Atrial	130
4	Accelerometer	Pig	Atrial	150
5	Accelerometer	Pig	Atrial	160
6	Accelerometer	Pig	Ventricular	120
7	Accelerometer	Pig	Ventricular	130
8	Accelerometer	Pig	Ventricular	150
9	Accelerometer	Pig	Ventricular	160
10	MRI tagging	Human	None	120

For the human heart motion dataset, the trajectory of the closest point to a preferred implantation site from a clinical point of view of the harvesting device was chosen. This point lies around 35 mm from the apex on the septal side, close to the midpoint between the apex and the pulmonary valve. The performance of the device was also investigated by placing it on the hexapod platform (Fig 3A) and then making the robot follow all ten trajectories. To analyse the dependency of the harvesting device’s spatial orientation with respect to gravity, the prototype was tested on different tilting angles on the hexapod’s platform. For each trajectory file (Table 1) the device was tested for orientation angles between -40° and 40° with incremental steps of 10° (Fig 3B–3D).

During the power output measurements, the voltage generated by the harvester was recorded in LabView (National Instruments Corporation, USA) via a data acquisition device (USB-6008, National Instruments, United States). The data was acquired for a period of 60 seconds with a sampling rate of 10 kHz and each measure was repeated 10 times. The voltage was measured over a resistance of 330 Ohms, matching the impedance of the generator coil to maximize the power output.

### VI. In-vivo study

The in-vivo experiment was performed on a 60kg domestic pig. Induction was performed with ketamin (20 mg/kg), xylazine (2 mg/kg) and atropin (0.04 mg/kg). The animal was then placed on the table in supine position and the trachea was desensitized with lidocain-spray, followed by endotracheal intubation. Inhalation anaesthesia was started and maintained with sevoflurane in oxygen (2.5–3% minimum alveolar concentration). The pig was purchased from a local breeder for large laboratory animals. The trial was approved by the Swiss Federal Food Safety and Veterinary Office and performed in compliance with the Guide for the Care and Use of Laboratory Animals of the National Research Council (US) Committee [17] (protocol number BE 84/16). The prototype was delivered by catheter. The pacing electrode was placed in the low right atrium for atrial pacing and in the right ventricular outflow tract (RVOT) for ventricular pacing.

The output power of the device was also measured over the optimal load resistance of 330 Ohms. The device was tested under the motion effect of the intrinsic heart rate of the animal as well as under artificial pacing in the right atrium and ventricle. The paced heart rates investigated were 120 bpm, 140 bpm and 160 bpm, for both pacing locations. Each measurement was performed once over a period of 3 minutes.

## Results

### I. Implant prototype

**Fig 4:**
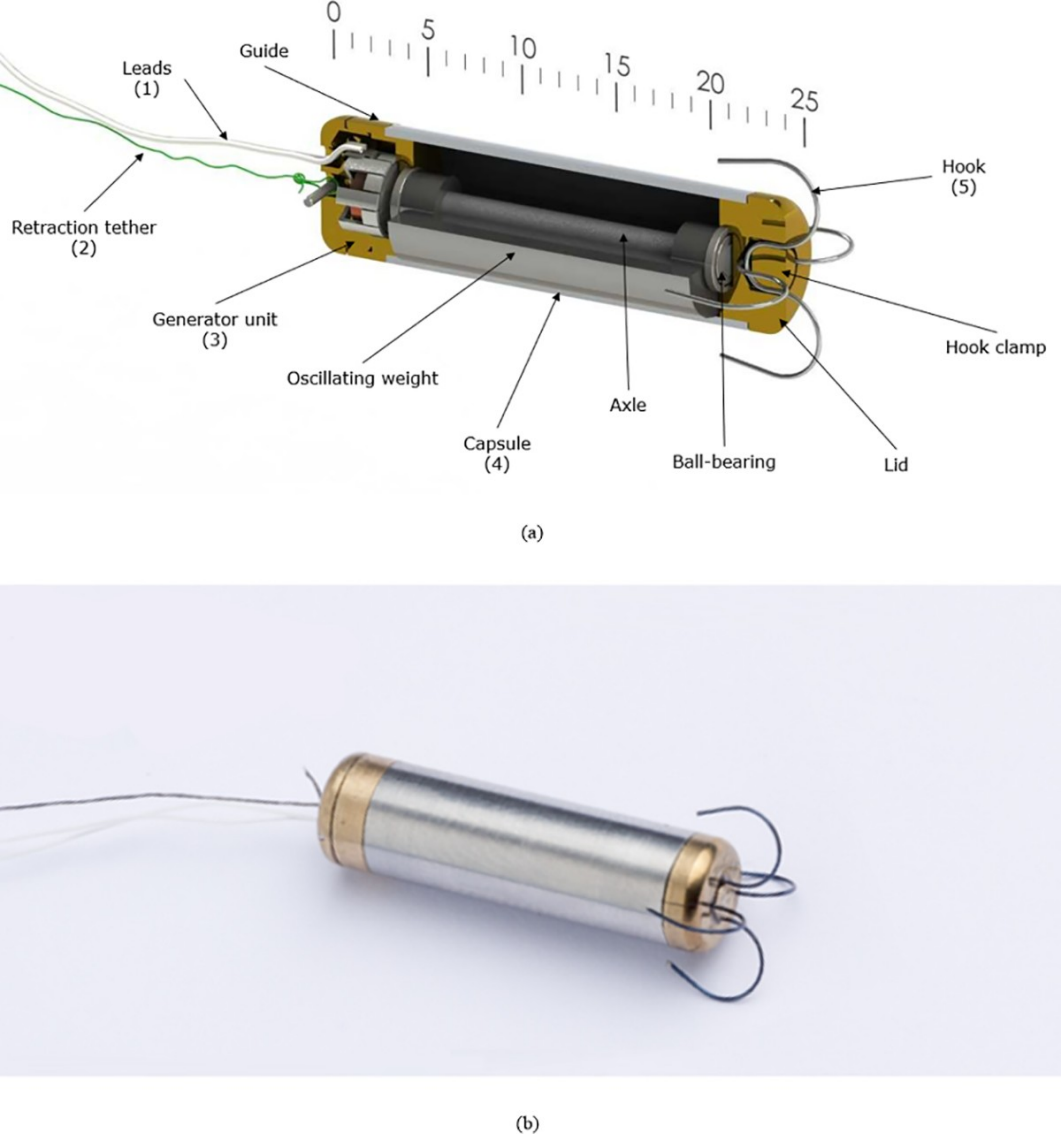
Cross-sectional view of the prototype (a). The prototype in its final 25 mm capsule form with a diameter of 6.8 mm (b).

The prototype (Fig 4) was designed in two main units: the power unit and the oscillation unit. Both units were integrated into a tubular housing (4). The capsule-shaped prototype has a length of 30 mm, a diameter of 7 mm, a volume of 1.15 cm^3^ and a total mass of 8.01 g. The oscillating weight is a half-cylinder made of platinum Pt960, with a diameter of 5.8 mm and a length of 16 mm. Through its center runs a steel shaft Ø1.5 mm, which is radially and axially suspended by two high precision ball-bearings (Fig 4A). The shaft end holds the electric generator’s rotor–a magnet with seven radially magnetized dipoles. The magnet is part of an electro-magnetic pole claw generator (Kinetron, The Netherlands) that converts the kinetic into electrical energy.

On the front end of the device, there are the nitinol hooks (5) for the fixation of the device on the endocardium. When the prototype is placed in the catheter, the hooks are bended in straight position with the tip directed on the opening of the catheter’s end. During the implantation procedure, the end of the catheter is positioned on the myocardium in a manner that when the prototype will be pushed out of it, the hooks will perforate the myocardium. The hooks will naturally retrieve their original form and fix the prototype to the myocardium. The hooks sit in a hole in the lid with their smallest radius. The hook clamp, a cylinder with a diameter of 2 mm, secures the hooks. All the different parts are fused and secured with glue. On the opposite side of the device, the generator unit (3) holds the outlet for the two electrical leads and the retraction tether. The electrical Ø0.1 mm wires are covered by biocompatible silicon and carry the electric signal from the generator out of the heart.

### II. Model validation

**Fig 5:**
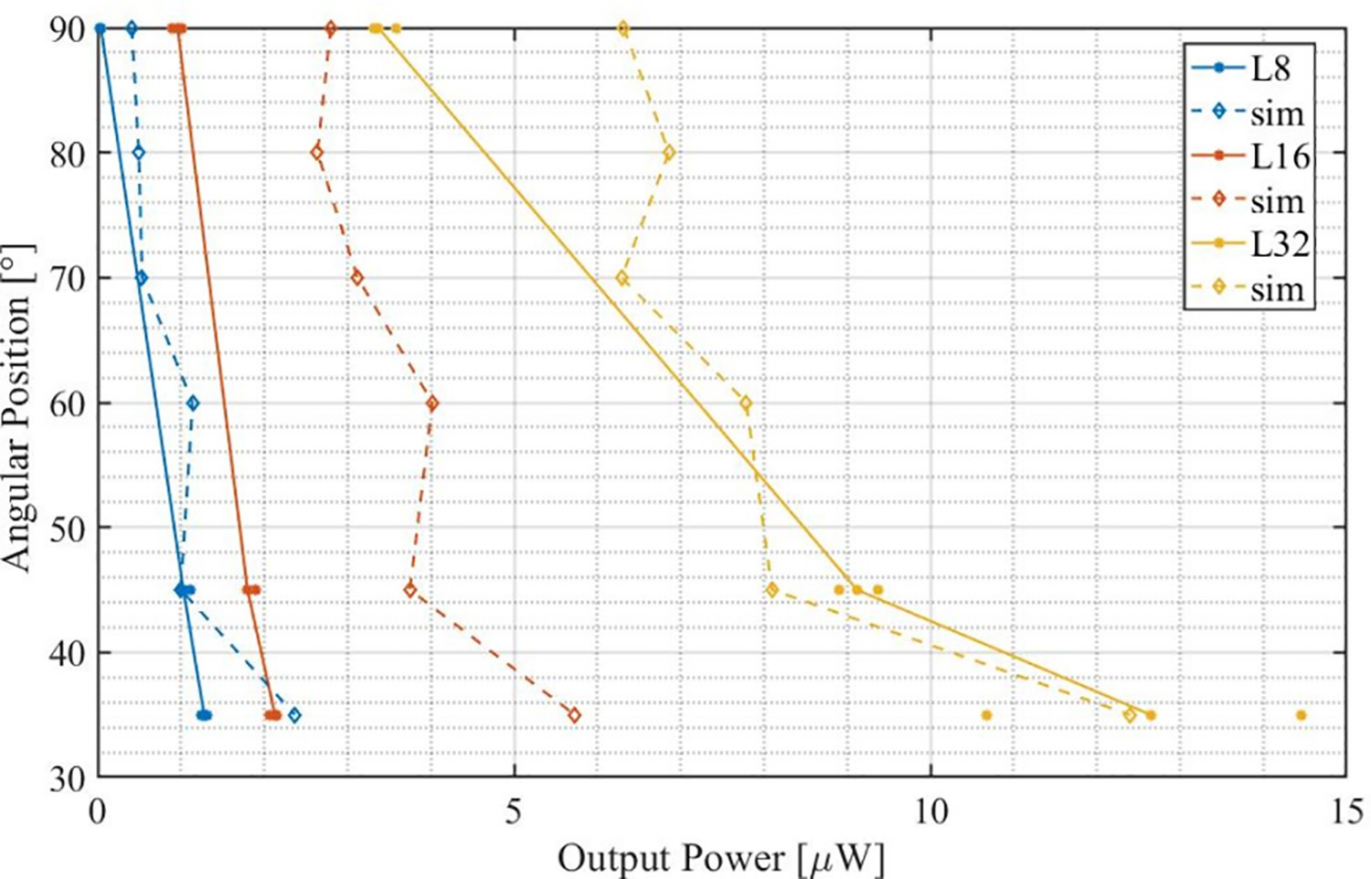
Results of the simulation (dashed line) in comparison to the output power measured during the experiment (continuous line) for 3 different weight length of respectively 8 (red), 16 (blue) and 32 (green) mm.

**Fig 6:**
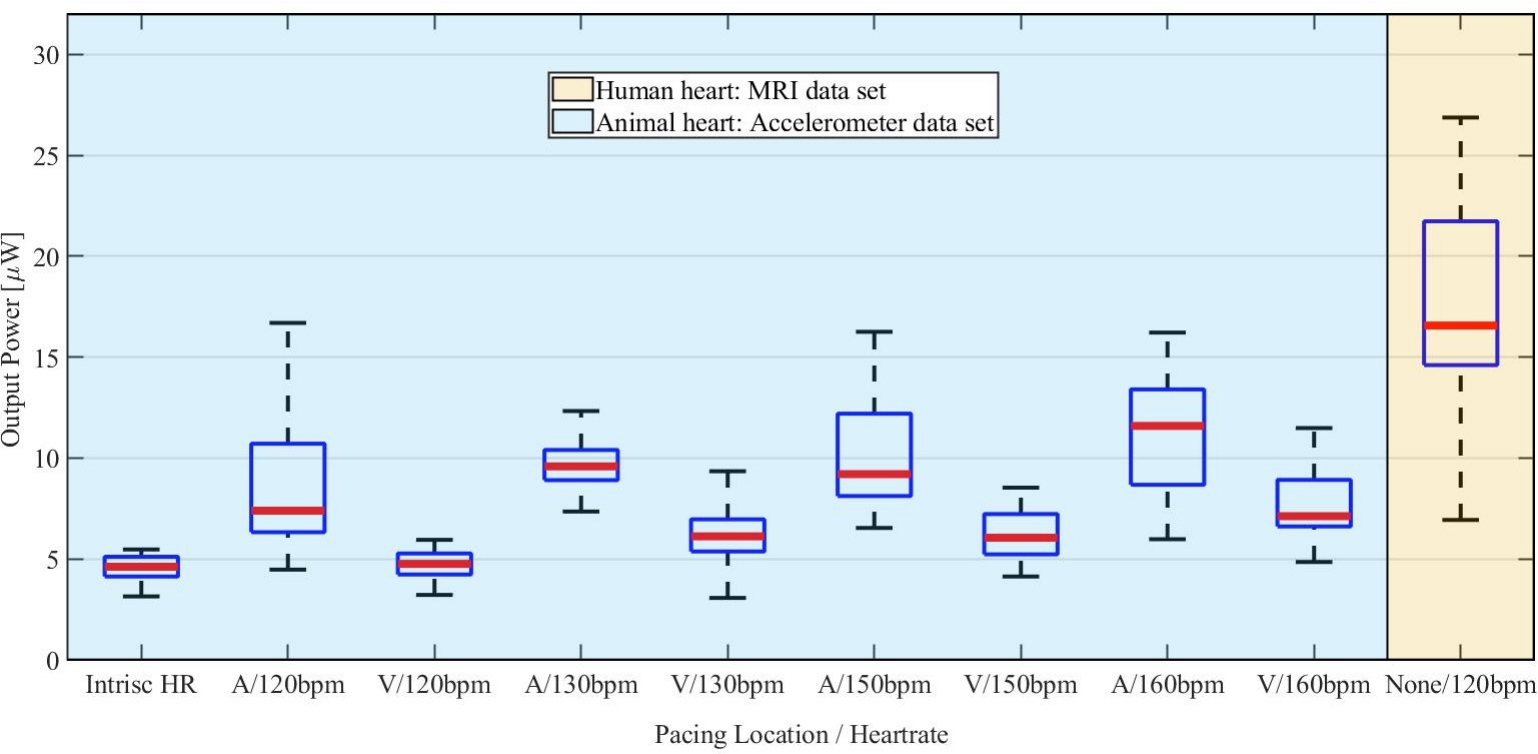
Average output power in function of the heart rate for the animal (blue) and human (yellow) datasets for atrial (A) and ventricular (V) pacing location.

The model estimated the power output for all tested oscillating weights the best when simulated with a damping coefficient of μ = 0.55 (Fig 6, dashed lines), compared to test bench experiments (Fig 5, solid lines).

Compared to the median measured output power, the simulated output power is overestimated by 43.7%, 59% and 21% for the weights L8, L16 and L32.

The results clearly indicate that better performance can be expected from heavier oscillating weights and supports the decision of using a high-density platinum weight in the final prototype. Simulations (partially) and measurements shows that the output power of the device decreases as its axis of rotation come closer to the gravity vector (orientation of 90°).

### III. Bench experiment on the hexapod

The median value of average output power measured with on all animal heart trajectories was 7.2 μW (IQR 5–11.4 μW) and the 95% confidence interval was ranging from 4.03 μW to 14.05 μW. 93% measurements lies above the target power required by actual endocardial pacemakers (4.2 μW). Fig 6 shows that the power generated by the prototype correlates positively with the heart rate. The median value of the measured output power was 4.6 μW (IQR 4.1–5.6 μW) for the intrinsic heart rate. The power increased to 5.9 μW (IQR 4.9–7 μW) for heart rates higher than 120 bpm (ventricular stimulation) and up to 9.6 μW (IQR 7.5–11.7 μW) for the same heart rates when atrial pacing was performed. The average output power measured with the dataset acquired on a human heart through MRI tagging is 16.6 μW (IQR 13.2–20 μW). Finally, the mean standard deviation of the measurements performed on the porcine heart trajectories for each tilt angle was ±0.6 μW and the absolute maximum standard deviation observed was ±1.5 μW.

**Fig 7:**
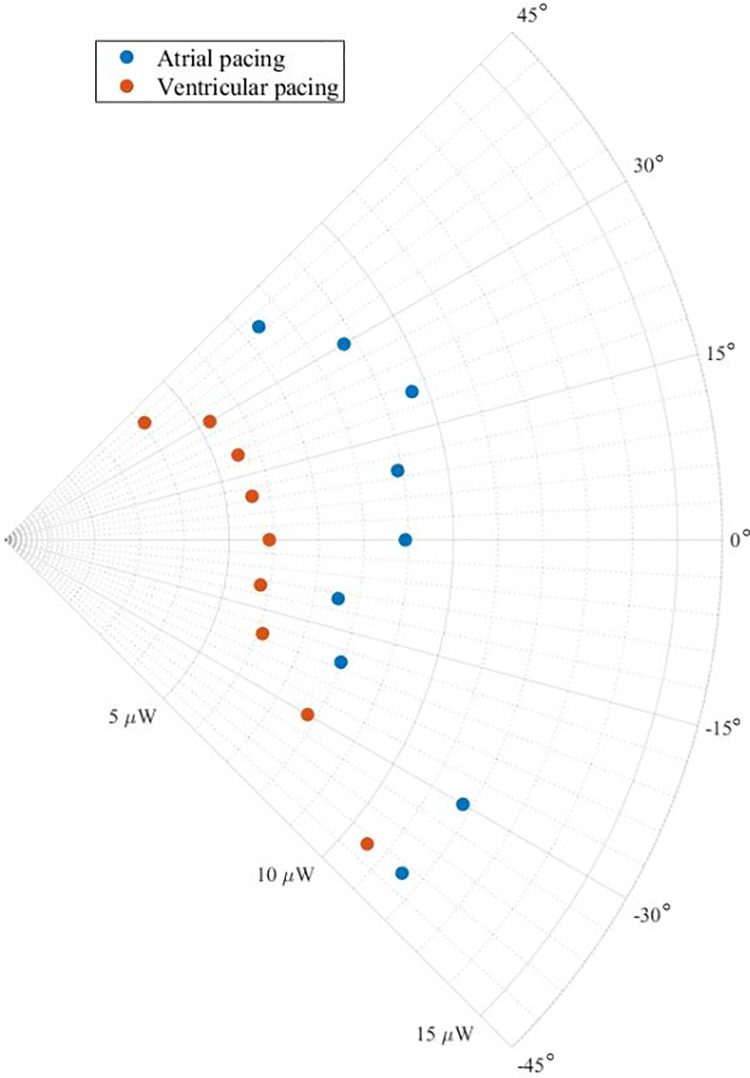
Average output power in function of the angular orientation (see Fig 3) of the harvester for atrial paced heart motion (blue) and ventricularly paced heart motion (orange).

Fig 7 shows the average output power measured at different tilting angles for an atrial or ventricular implantation site. Independent on the angle, the prototype consistently harvests more energy while the electric stimuli are delivered in the atrium. Under the effect of atrial paced heart motion, the mean measured output power of the prototype has a median value of 9 μW (IQR 4.2–13.8 μW). The mean value of the output power measured with the prototype undergoing the ventricularly paced heart motion is 6.1 μW (IQR 3.7–6.5 μW).

### IV. In-vivo study

**Fig 8:**
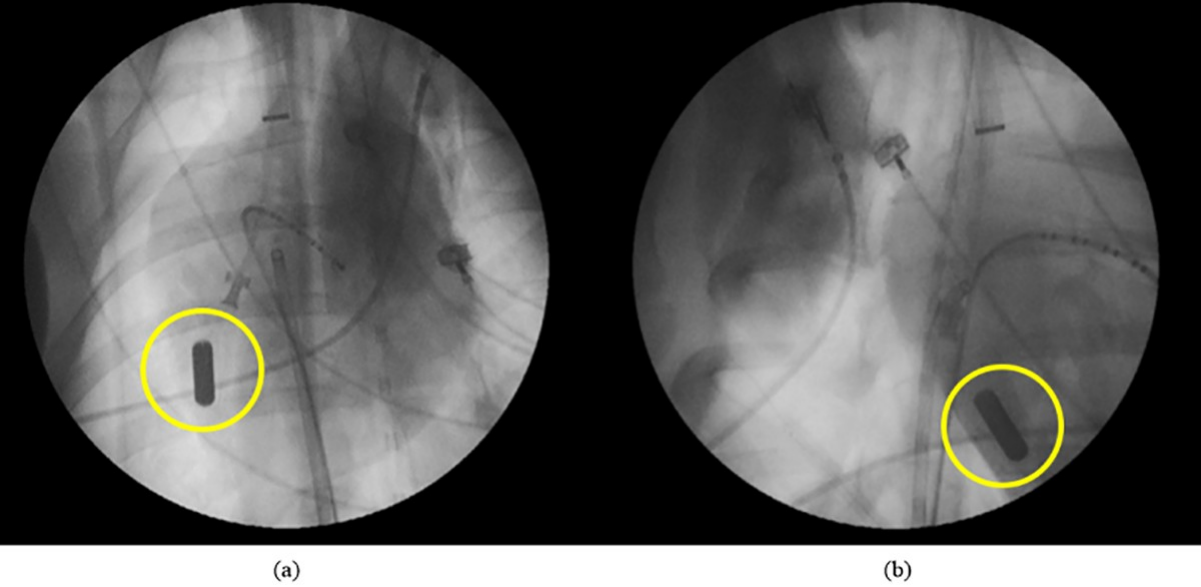
X-ray image from of the energy harvesting prototype inside a porcine heart during the in-vivo experiment. Panel (a) shows an antero-posterior view, panel (b) a right anterior oblique projection.

Our prototype was successfully implanted at the desired location–at an apico-septal location in the right ventricle (Fig 8).

**Fig 9:**
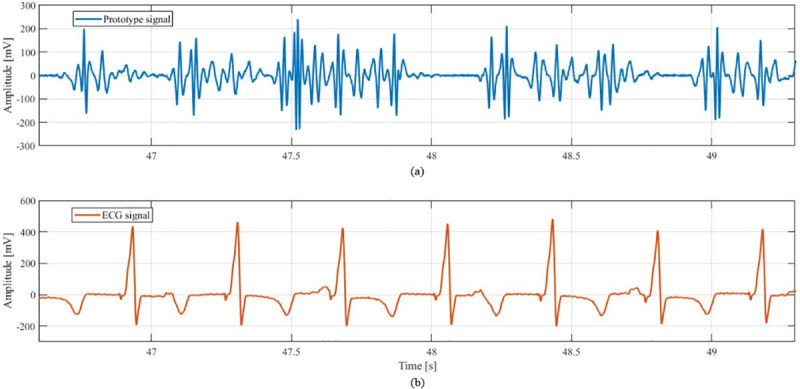
Example of the output Voltage of the prototype (a) and the corresponding ECG (b) when measured during the in-vivo study. This signal was acquired during ventricular pacing at a rate of 160 bpm.

Fig 9 shows a sample of the electric output signal of the device acquired during ventricular pacing at a rate of 160 bpm and the corresponding electrocardiogram (ECG).

An average output power of 2.6 μW was measured during the animal trial (Table 2). The average power was measured at 2.85 μW and 2.79 μW when atrial and ventricular pacing was performed, respectively.

**Table 2:** Average output power of the prototype measured during the in-vivo test.

Pacing Mode	Mean Heart Rate	Mean Output Power
	[bpm]	[μW]
None	89	1,13
AAI	120	1,38
AAI	142	2,43
AAI	163	4,73
VVI	120	1,4
VVI	137	3,26
VVI	160	3,71

## Discussion

Our energy harvesting prototype was estimated to generate a power output of 7.2 μW. The miniaturized prototype provided an average power of 4.2 μW and 2.6 μW during test bench and in-vivo experiments, respectively. The device was miniaturized to feature a volume of 1.2 cm^3^, which favours a catheter-based implantation in the right ventricle. This was successfully shown during a proof-of-concept in-vivo trial.

### I. Bench experiment on the hexapod

The heart motion was simulated with the aid of a robot reproducing heart motion trajectories acquired in-vivo. Fig 6 shows that the output power correlates with the heart rate and is above the power consumption of a contemporary pacemaker (4.2 μW) in 93% of the measurements performed on animal trajectories. The trajectory of a human heart induced an even higher overall output power. This may be related to anatomical and physiological differences between the human and porcine heart. Additionally, the MRI tagging process may also have contributed to this effect, as assumptions were made during acquisition and reconstruction of the trajectories [10].

As patients requiring PM implantation often suffer from cardiac diseases, impaired contractility would most likely also decrease an energy harvester’s output power. However, contraction abnormalities can be very localized, thus, not necessarily affect the energy harvester’s output negatively. For example, a localized dyskinesia at the left ventricular apex may lower overall ejection fraction but the movement of the harvest is still unimpaired as it is fixated on the interventricular septum. Widespread echocardiographic contractility measures such as the left ventricular ejection fraction, the fractional area change or even subjective heart failure grading schemes such as the New York Heart Association (NYHA) classification may not be directly suitable to estimate the output power of an endocardial energy harvesting system.

The bench-experiments demonstrated that the prototype could harvest a significant amount of energy. Importantly, the power output exceeds the target of 4.2 μW for all the orientations investigated in this study (Fig 7). This indicates that the presented energy harvesting mechanism robustly provides enough energy to supply future endocardial pacemaker for a wide range of angles.

Therefore, the energy output is unlikely to be affected significantly by posture changes of the patient. Comparatively, if the energy harvesting performances of piezo-electric transducers devices are promising [9, 13], there is a general lack of documentation on the effect on the harvesting performances of their orientation in the heart chamber. This information can have an important impact on the power generated by piezo-electric based devices where the mechanical-to-electrical transducer principle is based on a structural deformation of the material in one single-axis [18]. Thus, it is reasonable to suppose that the orientation of a vibrational energy harvesting device based on piezoelectric beam-based transducers will have a great impact on power output in-vivo. Similarly, electromagnetic transducers based on a one-axis moving part (magnets or coils) [12, 19] could also be affected by overestimation of the output power in an in-vivo application.

The harvested power was lower during ventricular pacing compared to atrial pacing (Figs 6 and 7). During sinus rhythm or atrial pacing, the electrical activation wavefront spreads physiologically from the atria through the His-Purkinje system into the ventricular myocardium, resulting in a highly coordinated activation of both ventricles. In contrast, ventricular pacing induced by artificial pacing is leading to mechanical dyssynchrony of the ventricles [20]. This abnormal activation reduces ventricular stroke volume and alters pressure-volume relationships [21]. These findings are in line with the reduced harvester power during ventricular pacing. This is also relevant from a clinical perspective as contemporary leadless pacemakers are intended to pace the heart in the right ventricle. This is of particular importance as the ideal candidates for leadless energy harvesting PMs would be younger patients with a high need of right ventricular pacing. Our investigation on the device’s orientation and pacing location dependency of the harvested power underline the importance that the estimated energy output in such patients is not to be overestimated based just on heart rate and a normal cardiac function. Although the described effect may vary with the harvester design, dependencies of the estimated power output on the stimulation pattern of the heart matter and were not considered by others so far [4, 6, 12]. The bench experiment also showed that the harvested power is well above the target power (4.2 μW) for higher heart rates. This highlights that especially young and non-sedentary patients are the most suitable candidates for endocardial energy harvesting pacemakers.

### II. Multiple energy sources for intra-body energy harvesting devices

Our energy harvesting mechanism relies on the constant heart contractions. However, our experimental setup as well as the in-vivo study does not consider real-life kinetic energy sources that would superimpose with the heart activity, e.g. body movement. For this reason, the harvester should provide the minimum power to supply the pacing circuit just by means of the heart motion. Additionally, accelerations from the heart superimpose with external accelerations and would most likely increase the overall amount of harvested power. However, the power harvested from other sources cannot just be added up to the mean power measured on the prototype when excited by artificial or natural heart motion. The reason is that the acceleration pattern of an external activity will be superimposed with the heart motion, mostly by increasing the external force vector that excite the oscillation weight. However, the superimposed acceleration can also inhibit the force vector if the acceleration and angular velocity effects of the walking movement are in the opposite direction of the heart’s one. Thus, the power estimate in the in-vivo animal test represents a conservative power output estimation during a resting state as the acceleration source is exclusively the cardiac motion.

### III. Prototype

The current prototype includes only 3 moving parts: the oscillating weight (with the corresponding shaft and the rotor magnet) and the two ball-bearings. Reducing the number of moving parts to the minimum was the main focus during the design process to minimize the effect of mechanical wear, which can lead to a reduction of the harvester performance over time. Although the volume of the protype is less than 1.2 cm^2^ and lies in the same range of actual endocardial PMs (<0.8 cm^2^), its actual weight (8 g) is four times larger and could have an impact on the heart. An in-vivo study presented in [22] was performed by suturing an inertial sensor carrying different additional loads on the epicardium of a porcine heart. The acceleration measurements showed an increase in ventricular wall acceleration with an increasing load. This led to the conclusion that that the cardiac muscle adapted to the additional load by increasing contractility, a well-known phenomenon in cardiac physiology (Frank-Starling mechanism). Nevertheless, as the prototype presented in this paper is meant to be implanted inside the heart, further in-vivo studies are necessary to describe the impact of its weight on the heart. Finally, in the current state, the energy harvesting prototype does not contain any integrated electronic or electric buffer which can potentially increase the device volume, mass, or limit the oscillating weight dimensions.

At the current development state of the prototype, the housing has a very functional purpose to facilitate device deployment by catheters used for implanting leadless pacemakers. Thus, the risks of complications and the cost of the surgery may be reduced compared to standard pacemakers and other energy harvesting prototypes [7–11]. Furthermore, if some of the other energy harvesting endocardial prototypes have similar [5] or even better [6] volume and mass (for a similar experimental set-up), the prototype presented in this paper has overall higher energy harvesting performance.

### IV. Mathematical model

The experimental setup demonstrated (Fig 5) how the mass (i.e. length) of the oscillating weight has a direct impact on the harvested power. Both simulation and measurements confirm, the heavier the oscillating weight, the larger the harvested power.

The experimental setup and the equivalent simulation analysis provide an estimation on how the tilt angle influences the output power (Fig 5). The output power decreases as the rotational axis of the pendulum aligns with the gravity vector, independently of the weight. When the rotation axis is not aligned with the gravity vector, the pendulum experiences a gravitational pole where it gets attracted to. For these cases, heart accelerations will excite the pendulum such that it will oscillate around the gravitational pole. Furthermore, in the special case that the rotation axis aligns with the gravity vector (90° tilt angle), the gravitational pole gets cancelled and the pendulum has no oscillation center anymore. This increases the chance that the oscillating weight stops at a position where it is aligned with the principal component of the external acceleration and may reduce the amount of rotation. However, this is a hypothetical case, as the attachment of the device on the myocardium is not rigid and body orientation is constantly changing with respect to gravity.

The numerical simulation provides an estimation of the generated output power (Fig 5). However, Fig 5 shows in some cases relevant differences between simulations and measurements. As the numerical model computes an exact numerical result for a given heart motion, the measurements of the bench experiments depend on how well the robot can mimic each individual heart motion. Therefore, measurements on the robot are bound to real-life constraints and may differ for different heart motions. For instance, a motion trajectory with high acceleration peaks is favourable for power generation but will create discrepancies with the simulation, when taking damping effects from mechanical and electronical set-up into account.

The damping force of the model includes the friction of ball bearings and the effect of air pumping by the oscillating weight in the enclosed housing. As these effects are difficult to measure on the bench, the damping coefficient of the numerical simulation was chosen such that its output matches the results of the test-bench experiments the best. Due to small oscillatory movement of the pendulum, gyroscopic effects were considered neglectable and therefore were not included in the model.

### V. In-vivo study

During the in-vivo study, the prototype harvested enough energy to supply a leadless pacemaker. It is crucial to note that the in-vivo data acquisition and the hexapod measurements are difficult to be compared directly:

- We did not observe a significant difference between the power measured under atrial or ventricular stimulation. The reason is likely that pacing the heart in the RVOT (as during the in-vivo trial) differs significantly from pacing from the left ventricular epicardial apex (as during the hexapod measurements). A different electrical activation pattern also leads to a different mechanical activation of the cardiac chambers.
- Furthermore, the in-vivo study and the experimental bench tests were not performed under the same conditions: the prototype was exposed to heart motion trajectories from a different animal than during the in-vivo trial. Despite both animals were chosen to have a similar age and weight, cardiac muscle morphology and physiology may have been different. However, with only one in-vivo test subject, the difference in the harvested power can also be caused by random effects and an in-vivo study including a large number of subjects would be necessary to statistically analyse the discrepancies between bench and in-vivo experiments.
- Additionally, since the heart contraction is mainly from apex to base, orienting the axis of rotation in parallel may decrease the harvested power. However, nowadays the preferred clinical implantation site of leadless PMs is more septal and not apical anymore [23], which will make the orientation of the device more perpendicular with respect to the direction of the contraction. This may increase the oscillation of the prototype’s weight.
- Finally, the hook’s geometry differs from actual endocardial pacemakers, which have a flat section and a shorter radius of curvature. These factors have an impact on the stiffness of the anchoring, which is lower for our prototype This may have led to a different heart-device-coupling and affect the transmission movement from the cardiac muscle to the device.

As a power supply for cardiac pacemakers, the harvesting device is supposed to remain on the ventricle for a long period of time. Therefore, chronical in-vivo trials will be required. First, the reason is to show the reaction of the heart to the additional load and furthermore, to investigate how the harvester efficiency is affected by potential changes of heart contractions. Finally, it is expected that the device will be at least partially encapsulated by surrounding tissue in a few month following the implantation [24, 25]. This can affect the energy harvester anchoring-tissue interface by increasing its stiffness leading to a better transmission of the movement from the muscle to the device.

### VI. Outlook on future challenge

This work does not consider an intermediate signal rectification and energy-storing circuit, which would have to be implemented for a fully functional batteryless endocardial PM. Although efficient solutions to rectify and store energy exist, there is no suitable electronic solution that efficiently convert the signal generated by this harvester, mainly because of its random oscillating behaviour and its low voltage amplitudes. For example, an active rectifier presented in [26] is able to rectify a 500 mV amplitude sinusoidal signal with 60% power conversion efficiency with a quiescent power consumption of 300 nW and the BQ25570 (Texas Instruments, USA) can boost voltage levels as low as 100 mV, with an efficiency varying from 20% to 70% and with a quiescent current consumption of 500 nA. Furthermore, the power consumption of an endocardial pacemaker can be more than 4.2 μW due to higher pacing thresholds (e. g. due to alterations of the electrode-tissue interface, or disease progression leading to a higher percentage of pacing). Such factors could potentially limit the number of patients suitable to be implanted with this kind of energy harvesting device.

Due to the use of a samarium-cobalt magnet for the generator rotor, the prototype will likely not be compatible with the high magnetic field necessary for MRI imaging techniques. However, permanent magnets made of high coercivity material such as Neodymium should be investigated, because of their high resistance to demagnetization.

Long term in-vivo trials will also be required to investigate how the harvester performance is affected by its potential encapsulation in the heart tissue. Mechanical degradation as well as how the additional weight (8 g without the electric circuit) might affect the heart must be investigated in a large in-vivo study. Furthermore, a chronical study will allow us to study the impact of daily life activities by measuring the power harvested by the prototype from all potential energy sources.

## Conclusion

Harvesting energy from the heart motion can eliminate the end-of-life limitation of today’s endocardial pacemakers to avoid multiple re-implantations. We demonstrated the feasibility of harvesting energy in the same range as the minimal power consumption of contemporary endocardial pacemakers (~4.2 μW) with a prototype in implantable shape and size-scale. Further optimization studies on the geometry, electromagnetic transducer mechanism as well as the development of a customized rectification circuit to supply a pacemaker can now be envisioned.
